# A novel ECG compression algorithm using Pulse-Width Modulation integrated quantization for low-power real-time monitoring

**DOI:** 10.1038/s41598-024-68022-5

**Published:** 2024-07-26

**Authors:** Isuri Devindi, Sashini Liyanage, Titus Jayarathna, Janaka Alawatugoda, Roshan Ragel

**Affiliations:** 1 Department of Computer Engineering, University of Peradeniya, Peradeniya, Sri Lanka; 2 The MARCS Institute for Brain, Behaviour and Development, Western Sydney University, Westmead, NSW Australia; 3 Research and Innovation Centers Division, Rabdan Academy, PO Box 114646, Abu Dhabi, United Arab Emirates; 4 Institute for Integrated and Intelligent Systems, Griffith University, Nathan, QLD 4111 Australia

**Keywords:** ECG compression, Hybrid PWM, Low-complexity, Nearly-perfect reconstruction, Real-time algorithm, Biomedical engineering, Cardiac device therapy

## Abstract

Cardiac monitoring systems in Internet of Things (IoT) healthcare, reliant on limited battery and computational capacity, need efficient local processing and wireless transmission for comprehensive analysis. Due to the power-intensive wireless transmission in IoT devices, ECG signal compression is essential to minimize data transfer. This paper presents a real-time, low-complexity algorithm for compressing electrocardiogram (ECG) signals. The algorithm uses just nine arithmetic operations per ECG sample point, generating a hybrid Pulse Width Modulation (PWM) signal storable in a compact 4-bit resolution format. Despite its simplicity, it performs comparably to existing methods in terms of Percentage Root-Mean-Square Difference (PRD) and space-saving while significantly reducing complexity and maintaining robustness against signal noise. It achieves an average Bit Compression Ratio (BCR) of 4 and space savings of 90.4% for ECG signals in the MIT-BIH database, with a PRD of 0.33% and a Quality Score (QS) of 12. The reconstructed signal shows no adverse effects on QRS complex detection and heart rate variability, preserving both the signal amplitude and periodicity. This efficient method for transferring ECG data from wearable devices enables real-time cardiac activity monitoring with reduced data storage requirements. Its versatility suggests potential broader applications, extending to compression of various signal types beyond ECG.

## Introduction

Electrocardiogram (ECG) analysis is widely recognized as the primary clinical cardiac test used by healthcare professionals for screening various cardiac abnormalities due to its low-risk, cost-effective, and straightforward application. Long-term monitoring of the electrical activity of the heart for early detection of transient or infrequent arrhythmias is challenging without computer-aided diagnosis methods.

Over time, many sophisticated and highly accurate computer-aided arrhythmia diagnosis methods have been introduced for short-term monitoring (< 30 min) of the ECG signal^1^. Holter monitors^2^ are wearable devices specifically designed to monitor the ECG in real-time for a longer period. With the introduction of wearable devices such as smartwatches, the possibility of real-time heart disease detection is now made available to a wider community. Signals monitored over a long period often require a lot of storage and transmission bandwidth. Wirelessly connected cardiac monitoring systems connected to Internet of Things (IoT) healthcare platforms are usually battery-driven, have limited storage, and have a minimum computational capacity^1,3^. It is essential to have minimum local processing and wirelessly transfer vital information to cloud-connected servers with high computational resources for further analysis.

Wireless transmission is one of the power-hungry parts of an IoT device^3^. Therefore, compression of ECG signals is essential for reducing the amount of data transmitted. The existing compression algorithms fall under two main methods: lossless and lossy compression^4^. Lossless ECG compression algorithms do not alter the amplitude and periodicity details of the signal, but they are not suitable for real-time applications due to their high complexity^1,3–5^. On the other hand, lossy compression techniques can achieve high compression ratios, but they are less reliable when compared to lossless techniques due to the introduction of distortions or artefacts that can affect the quality and accuracy of the analysis^3–5^. In particular, it is necessary to preserve features such as heartbeat intervals and wave morphologies of the different ECG components, such as the P, QRS, and T waves, that contain vital information about cardiac activity and rhythm. The compression method should allow for accurate QRS detection, which is the basis for many applications such as arrhythmia and heart rate variability detection, but has not been addressed in most existing works^3,4,6^. Therefore, a simple, fast, efficient data compression technique that consumes negligible power, time, and minimum storage is necessary to reduce the high volume of data generated by long-term ECG monitoring while preserving the quality of the signal for further processing.

In this paper, we introduce a novel, simple compression method that builds upon standard quantization. This method adjusts the quantized data point values based on the accumulated quantization error. The outcome is a hybrid Pulse-Width Modulated (PWM) signal, which retains the original signal shape at a low quantized level and represents the accumulated error in PWM in the same waveform. The resulting signal can be easily restored using a low-pass filter. To assess the performance of this compression algorithm, we used conventional signal quality metrics like Bit Compression Ratio (BCR) and Percentage Root-Mean-Square Difference (PRD). The proposed algorithm is also tested against application-oriented measurements such as QRS detection and heart-rate variability measurements. Despite its low computational complexity, which minimizes the use of multiplications and additions and avoids floating point operations, the algorithm effectively maintains QRS peak detection and heart-rate variability measurements. This indicates that the algorithm preserves both the signal’s amplitude and periodicity without compromising arrhythmia detection analysis accuracy.

**Figure 1:**
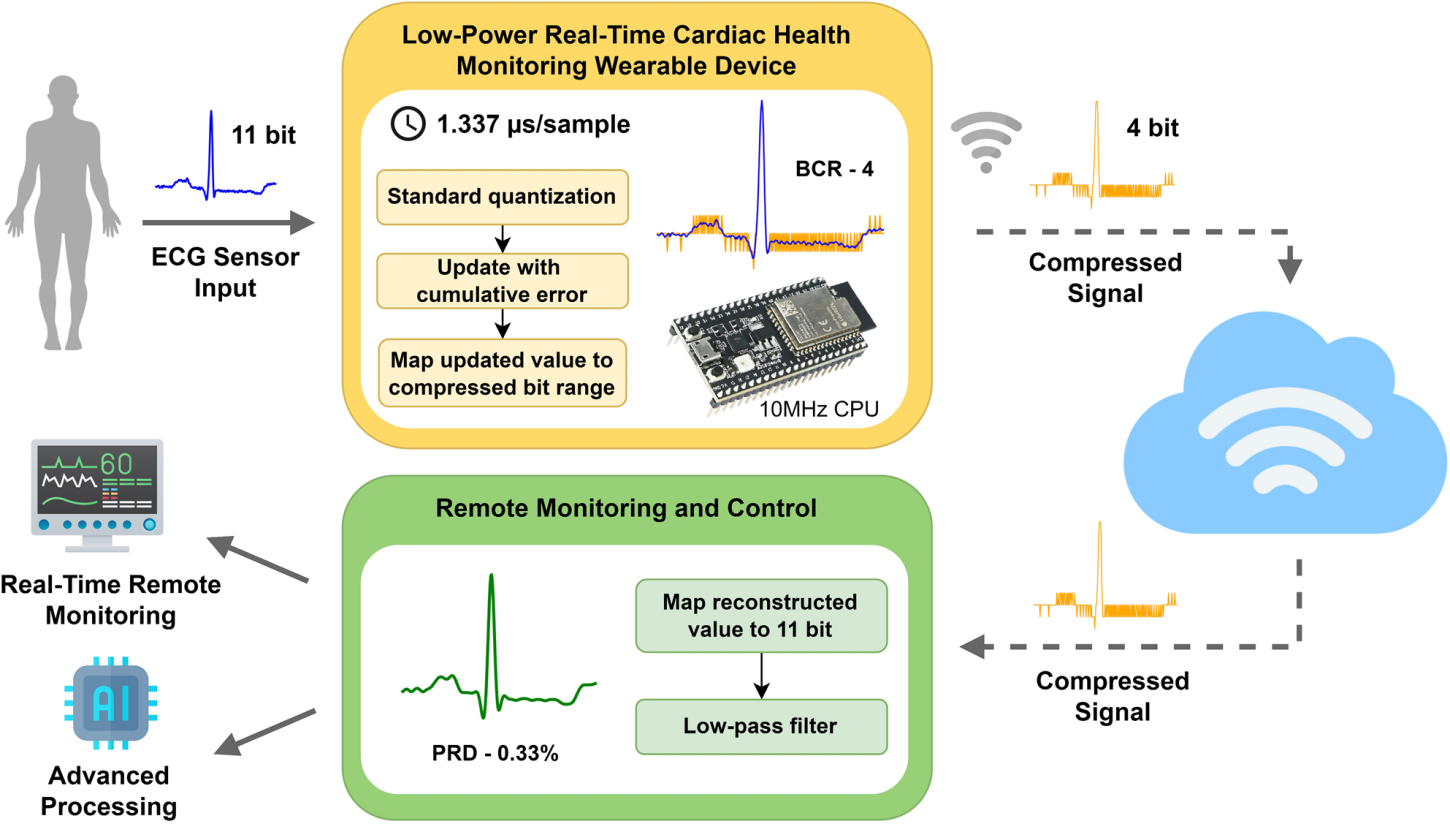
Real-time low-power cardiac health monitoring system.

In scenarios such as tele-cardiology and ambulatory recording systems, battery-driven monitoring devices continuously record ECG data in real-time. These devices either store the ECG data and transmit it to the healthcare center once the recording period is complete or transmit the data in real-time when an alert signal is detected. Typically, these devices have sampling rates ranging from 100 to 1000 Hz with a resolution of 8 to 16 bits per data sample^5,7^. For instance, a 24-hour recording of an ECG signal with a 360 Hz sampling frequency and 11-bit sample resolution generates approximately 40.8 MB of data per lead. This results in a substantial volume of ECG data collected during long-term cardiac monitoring. The increasing data size necessitates more storage space, higher bandwidth, extended transmission time, and reduces battery life. Reducing the data size lowers memory requirements for storage and transmission time, thereby decreasing energy consumption and extending battery life. The optimized solution proposed in this work is designed for use in battery-operated ECG devices that collect and transmit ECG signals in real-time. As illustrated in Fig. 1, the algorithm is capable of running on a low-end processor with a lower operating frequency. It efficiently compresses high-resolution ECG sensor data in real-time, reducing the data to a smaller number of bits with negligible distortion. This compression significantly reduces transmission delays, preserves bandwidth, saves energy, and minimizes memory usage and power consumption. Consequently, it is ideal for real-time, long-term monitoring, enabling continuous and efficient remote cardiac monitoring and analysis.

The remaining part of the paper is organized as follows: The related work section provides a summary of existing work on ECG signal compression. The method section contains the full description of the proposed ECG compression algorithm. Performance evaluation along with the results are presented in the results and discussion section before concluding the paper.

## Related work

Due to the large amount of data generated by continuous ECG monitoring, compression techniques have evolved to efficiently store and transmit ECG data while minimizing the impact on diagnostic information. Over the past few decades, numerous ECG compression methods have been introduced. These existing ECG compression techniques can be categorized into three main groups: transform-domain, direct time-domain, and parameter extraction approaches.

### Transform-domain approaches

Transformation-based compression techniques for ECG data often involve utilizing linear orthogonal transformations on ECG samples. Thus, the compression takes place in a completely new domain, such as the Fourier Transform (FT) domain, Discrete Cosine Transform (DCT) domain, and Wavelet Transform (WT) domain. During signal reconstruction, an inverse transformation is applied to recover the original signal with a certain degree of error. These techniques typically offer a higher BCR compared to direct time-domain compression methods and are robust to noise that may be present in ECG signals^8–12^.

Shinde and Kanjalkar^13^ have documented observations in Table 1 regarding Signal-to-Noise Ratio (SNR), PRD, and BCR as part of their comparison of different transform-based methods for ECG data compression.

**Table 1:** Comparison of different transform domain techniques in terms of Percentage Root-Mean-Square Difference (PRD), Signal-to-Noise Ratio (SNR), Bit Compression Ratio (BCR).

Transforms	Advantage	Disadvantage
FFT	Good PRD and SNR	Low BCR
DCT	Improved BCR compared to FFT	Lower SNR and increased PRD than FFT
WT	High BCR while maintaining low PRD	Lower SNR than FFT

Transform-based compression techniques often have high computational complexities and complex mathematical operations^14^ that require significant computational resources, including floating-point units, memory, and processing power, which can quickly drain limited battery life, making them impractical for real-time scenarios with constrained hardware^15^.

Recently, there has been significant development of low-complexity ECG compression techniques^16,17^ to meet the specific needs and constraints of modern healthcare applications. However, some low-complexity algorithms still employ thousands of mathematical operations per sample that result in higher computational overhead^16^. Some research publications lack a clear specification of the required computations^17^, which makes them unsuitable for real-world plug-and-play solutions without testing the resource consumption.

### Direct data compression methods

Direct data compression methods generally utilize prediction or interpolation algorithms and do not perform preprocessing as in transformation-based methods. Different direct data compression methods are Amplitude Zone Time Epoch Coding (AZTEC)^18,19^, Coordinate Reduction Time Encoding System (CORTES)^20^, Turning Point (TP)^21^, SLOPE^22^, and Fan and Scan-Along Polygonal Approximation (Fan/SAPA)^23^ algorithm. A summary of these can be found in Jalaleddine et al.^24^ and Kumar et al.^4^. These compression methods introduce higher PRD and are unsuitable for resource-constrained applications due to their complexity.

Nowadays, research is focusing on direct compression real-time techniques that are optimized for resource-constrained environments rather than aiming at the higher compression ratios provided by transform-based methods^1,25–27^. However, one notable shortcoming is the absence of a demonstration of how the reconstructed ECG signal impacts QRS detection and beat classification, particularly in terms of preserving essential features. Moreover, the delays and phase shifts introduced by the low-complex methods^25^ might not be suitable for wearable devices where real-time processing is required.

### Parameter extraction methods

Parameter extraction techniques in ECG compression aim to identify and encode specific signal features, like the location of maxima and minima points of heartbeats, slope changes, and zero crossing intervals, which contain the most critical diagnostic information. The process of parameter extraction is irreversible and involves isolating specific characteristics or parameters from the signal, which are then utilized for compression based on prior knowledge of the signal. Features most commonly used in parameter extraction compression methods are linear prediction, vector quantization, neural networks^28^, peak picking^29^, and long-term prediction^30^.

However, many of these parameter extraction methods involve computationally intensive tasks, such as QRS complex detection, waveform delineation, and heart rate variability analysis, before using these parameters for compression. Executing these operations on noisy ECG signals is prone to errors, which may result in significant distortions in the compressed signal. Using reconstructed signals for future processing and monitoring can be harmful.

Most of the existing methods have not adequately addressed the reduction of floating-point arithmetic to mitigate hardware requirements^28^. Floating-point hardware is complex because it is designed to achieve a wide dynamic range and high precision. This complexity makes the hardware more resource-intensive and potentially slower than simpler integer or fixed-point hardware. Therefore, in low-complexity, resource-constrained environments, integer or fixed-point arithmetic is the preferable choice.

The existing work’s main drawbacks include:

- Transform domain approaches offer better compression with less distortion but are unsuitable for real-time compression due to their complexity.
- Direct data compression techniques, although less computationally demanding, involve floating-point operations, necessitating complex hardware. They also introduce delays and phase shifts to the signal.
- Parameter extraction methods struggle to extract prominent features from noisy signals, leading to distortions in compressed signals.

The proposed algorithm addresses these issues by employing a simple fixed-point operation-based approach that is resistant to signal noise, resulting in minimal distortion, delays, and phase shifts, making it suitable for real-time compression.

## Method

In this section, we discuss the methodology of our research in detail.

### Datasets

In practice, the sampling rates of ECG acquiring systems vary from 100–1000 Hz, with a resolution of 8–16 bits per data sample^5,7,25^. Therefore, two databases with data in different resolutions and sampling rates were used for the experiments and performance evaluations.

The widely recognized MIT-BIH arrhythmia database^31,32^, which includes 48 half-hour ambulatory ECG recordings sampled at 360 Hz, with two channels (lead-I and lead-II), was used to assess the proposed compression algorithm’s effectiveness. The digitized lead-II recordings obtained via the WaveForm DataBase (WFDB) Software^33^, with an 11-bit original resolution, was used to test our compression algorithm. Lead-II is commonly used in arrhythmia diagnosis and monitoring due to its clear and dependable representation of the P-wave and QRS complex amplitudes.

The INCART database (St.-Petersburg Institute of Cardiological Technics 12-lead arrhythmia database)^31^, containing 75 recordings of 30 min each sampled at 257 Hz with a 16-bit resolution, was used to validate the algorithm’s performance.

The methodology and primary results, discussed in the subsequent sections, focus on the MIT-BIH database as the majority of work summarized in Table 2 has used it. To verify the algorithm’s robustness, the INCART database was used, and the results are discussed in a separate subsection in the results and discussion section.

**Table 2:** Compression performance comparison with other algorithms in terms of Bit Compression Ratio (BCR) and Percentage Root-Mean-Square Difference (PRD).

Type	Method	Performance		Comments	
		BCR	PRD (%)	Advantages	Disadvantages
Transform-domain approaches	Quantized DCT coefficient^8^	9.3	2.5	Higher CR	Complex Higher PRD
	USZZQ and Huffman coding^9^	11.06	2.73		
	Wavelet-based hybrid data compression^10^	18	2.6		
	Empirical mode decomposition and wavelet transform^11^	21.56	6.80		
	Empirical mode decomposition and tunable-Q wavelet transform^12^	33.11	4.35		
	Wavelet Packet (WP) technique^16^	7.17	1	Low PRD	Complex
	(N-PR) Cosine-modulated filter bank^16^	8.13	1		
Direct data compression	Improved AZTEC^19^	2.76–9.91	4.54–7.99	Less sensitivity to noise Relatively less complex than frequency-domain methods	Complex for resource-constrained applications Higher PRD
	CORTES^20^	4.8	7.0		
	TP^21^	2	5.1		
	SLOPE^22^	4.8	7		
	Fan/SAPA^23^	3	4		
	Cross-channel predictor compression^25^	2.92–3.43	0	Lossless	Higher computations per sample
	Joint coding packaging scheme^26^	2.38	0		
	Huffman data compression algorithm^27^	2.43	0		
	Adaptive linear predictors with decimation compression^1^	6	1.88	Simple	Low quality score (CR/PDR)
	**Proposed algorithm**	**4.0**	**0.33**	**Simple nearly perfect reconstruction**	**Higher CR comes with higher PDR**
Parameter extraction	Deep convolutional autoencoders^28^	32.25	2.73	Higher CR	Complex
	Adaptive sampling on generalized perceptual features^29^	3.0–4.7	3–5	–	

Performance of the proposed algorithm are in bold.

### Proposed ECG compression algorithm

**Figure 2:**
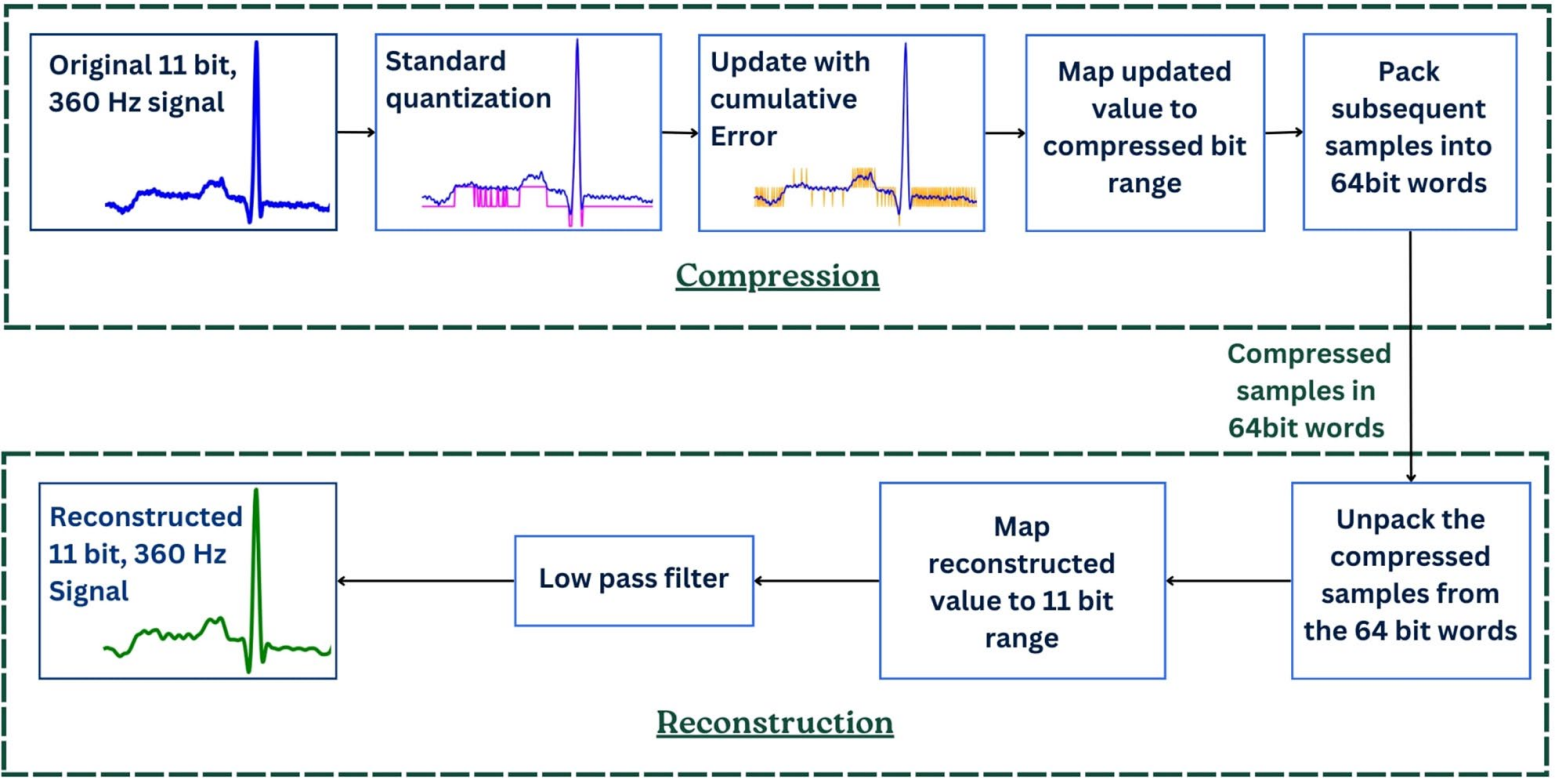
The proposed algorithm structure.

A novel compression algorithm built upon the standard quantization algorithm is proposed by manipulating the quantized values to produce a signal with varying pulse width using the steps shown in Fig. 2. Each of the sample points in the original signal in the range [0, 2047] (11-bit resolution) is initially quantized to be represented using a desired number of compressed bits. This quantization is achieved by dividing the original value by a chosen step size as shown in Algorithm 1.

**Algorithm 1:**
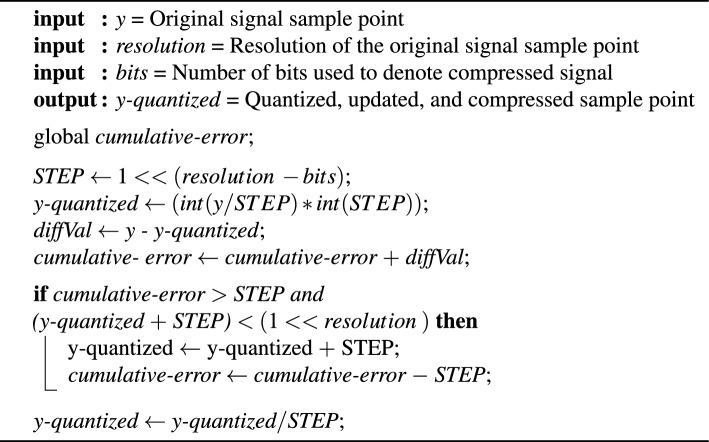
Compression Algorithm

Then, the quantization error is accumulated over the sample points. To generate a PWM-like signal, the step size is considered as the reference point. When the accumulated error is less than or equal to this step size, the quantized value remains unchanged, resulting in a PWM pulse of extended width. On the other hand, when the accumulated error exceeds the step size, the quantized value is updated by adding an amount equal to the step size, resulting in a pulse with a shorter width.

**Figure 3:**
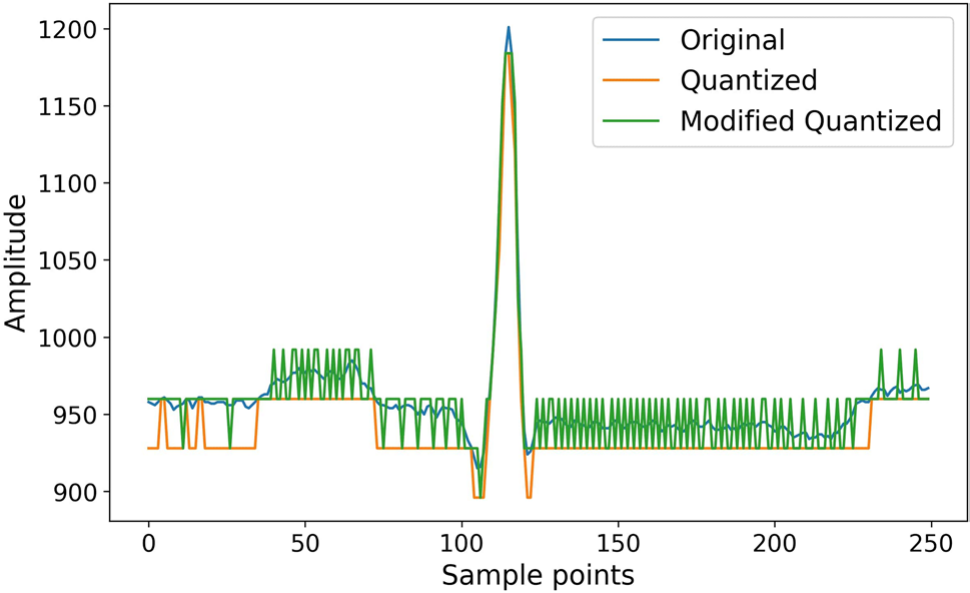
The difference between the original, quantized, and signal generated by modifying the quantized signal for 6 bits compression.

The purpose of such an update is twofold: (I) to reduce the cumulative error of quantization and (II) to ensure a more accurate reconstruction by encoding the information about the quantized values into the pulse width, creating a signal that exhibits characteristics similar to a PWM signal. The difference between the original, quantized, and signal generated by modifying the quantized signal is shown in Fig. 3 for 6-bit compression.

The compressed signal is generated from the quantized signal by assigning each quantized value to a specific range determined by the desired number of bits. This is done by dividing the quantized value by the step size.

### Reconstruction of the compressed signal

Since the compressed signal is stored in a lower resolution chosen by the number of bits, the compressed signal is multiplied by the step size to obtain a signal in 11-bit resolution. Afterwards, the reconstruction of the compressed signal is done by passing the signal through the Butterworth low-pass filter, as shown in Fig. 2.

The cumulative error stored in the PWM-like signals can be transformed back into the original signals using a simple RC-type low-pass filter. The duty cycle determines the magnitude of the filter’s voltage output. As the duty cycle increases, the average voltage output increases, and vice versa. A 3rd-order Butterworth low pass filter with a cut-off frequency of 27 Hz is used. Butterworth filters have a maximally flat frequency response in the passband, introducing minimal distortion to the signal within the desired frequency range. A filter of order three is used to achieve a satisfactory roll-off rate while minimizing the overshoot and ringing effect. The reason for a cut-off frequency of 27 Hz is to reconstruct the signal to maximize the QRS energy that lies in the range of approximately 5–15 Hz while reducing the influence of muscle noise, 60 Hz interference, T-wave interference, and baseline wander^34^. The transfer function of the low-pass Butterworth filter is shown in Eq. (1). The reconstruction of the compressed signal is done according to Eq. (2).

1 $$\begin{aligned} H(s)= & {} \frac{1}{1 + \left( \frac{s}{j\omega _c}\right) ^{2N}} \end{aligned}$$

2 $$\begin{aligned} F(s)= & {} H(s) \cdot PWM(s) \end{aligned}$$

Where:

- F(s) is the reconstructed signal,
- PWM(s) is the compressed signal,
- $j$ is the imaginary unit ($j^2 = -1$),
- $\omega _c$ is the angular cutoff frequency ($\omega _c = 2\pi f_c$),
- $N$ is the order of the filter.

### Resampling for further compression

Experiments were also carried out to further increase the compression by down-sampling the compressed signal of 360 Hz sampling rate obtained from Algorithm 1. Down-sampling is achieved by a simple linear interpolation. This technique was used to reduce the number of sample points saved per recording. Two different sampling rates were identified as 180 Hz and 120 Hz, reducing the number of stored points per recording by half and ${ 1/3 ^{rd}}$, respectively. The reconstruction of a down-sampled signal is done using the same steps discussed in the previous section.

### Storing the compressed signal

Each recording of the compressed signals is stored as 64-bit word binary files by packing multiple sample points in a single 64-bit word to achieve maximal compression.

### Testing platform

Experiments were carried out using the LOLIN32 Lite, a cost-effective ESP32 board. Similar to other ESP32 boards, the LOLIN32 Lite features a 32-bit dual-core microcontroller equipped with 4 MB of flash storage and 520 KB of RAM.

The algorithm was implemented in the C programming language. The sensor data was serially transmitted to the microcontroller via the UART module, and the proposed compression algorithm was run for each sensor data point received at a frequency of 360 Hz.

The time and power consumptions were measured at various CPU clock frequencies, specifically at 240, 160, 80, 40, 20, and 10 MHz. The power consumption to run the algorithm was measured by measuring the current and voltage across a 1.1$\Omega$ resistor connected serially to the ground of the microcontroller as shown in the ground resistor method in^35^ using the MP730026 hand-held digital multimeter.

### Evaluation metrics

The reconstruction signal quality is evaluated using the parameters below, which take compression efficiency and error into consideration.

#### Percentage root-mean-square difference (PRD)

Measures the error between the reconstructed and original signal.

3 $$\begin{aligned} PRD = \sqrt{\frac{\sum _{n=1}^{N} [x(n)-{\hat{x}}(n)]^{2}}{\sum _{n=1}^{N} [x(n)]^{2}}} \cdot 100 \end{aligned}$$

Where:

- *N* is the number of data samples,
- *x*(*n*) is the original signal,
- ${{\hat{x}}(n)}$ is the reconstructed signal.

#### Bit compression ratio (BCR)

The compression efficiency is measured using the bit compression ratio (CR), which is given as,

4 $$\begin{aligned} BCR = {\frac{N_o}{N_r}} \end{aligned}$$

Where:

- $N_o$ is the number of bits required to represent the original signal,
- $N_r$ is the number of bits required to represent the compressed signal.

The BCR provides information on the degree of redundant data removal. Higher BCR leads to a lesser number of bits required to store and transmit data.

For the purposes of this study, we assume that a single sample of the original 11-bit data is stored as 16 bits (2 bytes) in a byte-aligned system.

#### Quality score (QS)

Quality Score quantifies how accurately the compressed ECG signal represents the original signal while reducing the amount of data required to represent an ECG signal while minimizing the loss of clinically relevant information.

5 $$\begin{aligned} QS = {\frac{BCR}{PRD}} \end{aligned}$$

Where:

- *BCR* is the Bit Compression Ratio,
- *PRD* is the Percentage Root-Mean-Square Difference.

#### Space saving

The amount of space saved by compressing a file or folder containing multiple files is given as,

6 $$\begin{aligned} Space\;Saving = \left( {1- \frac{F_r}{F_o}}\right) \times 100\% \end{aligned}$$

Where:

- $F_r$ folder size after compression,
- $F_o$ folder size before compression.

#### QRS detection performance

While the evaluation of current literature is widely done by PRD, it is vital to report the impact of compression on detecting QRS complexes as well. Since a highly distorted reconstruction that does not preserve important features of the signal can be futile, the validity of the proposed compression technique is evaluated on QRS complex detection.

This evaluation utilizes popular ECG R-peak detection algorithms, namely Pan-Tompkins, Elgendi, Hamilton, and XQRS, obtained from py-ecg-detectors^36^ and WFDB^33^ respectively. For this comparative analysis, 44 out of the 48 MIT-BIH arrhythmia ECG records were utilized, excluding records 102, 104, 107, and 217 due to the presence of pacemaker signals^37^.

For a fair and consistent evaluation of the proposed method with all results published in the literature, the performance of the QRS detection algorithms from each method was assessed using two statistical measures: sensitivity (SE) and positive precision (+P) are calculated as follows,

7 $$\begin{aligned} SE= & {} \left( {\frac{TP}{TP+FN}}\right) \times 100\% \end{aligned}$$

8 $$\begin{aligned} +P= & {} \left( {\frac{TP}{TP+FP}}\right) \times 100\% \end{aligned}$$

Where:

- *TP* is the number of QRS complexes detected as QRS complexes (True Positive),
- *FP* is the number of non-QRS complexes detected as QRS complexes (False Positive),
- *FN* is the number of QRS complexes which has been missed (False Negative).

SE given in Eq. (5) reports the percentage of true beats detected out of all true beats, whereas +P in Eq. (6) reports the percentage of correctly detected beats out of all detected beats.

#### Heart-rate variability performance

The evaluation of heart-rate variability involves the computation of the average R-peak to R-peak (R-R) distance difference between the original and reconstructed signals. The XQRS R-peak detection algorithm, chosen as the most effective among the four algorithms considered in this study, is used to identify the R-peaks and an average R-R distance is determined for each minute of the signal. This is done by considering a sampling frequency of 360 Hz over a 60-s duration ($360\times 60$ samples). The error for each minute is then calculated as the difference between the average R-R distances. The average of these errors represents the overall heart-rate variability measurement. Mathematically, this can be expressed as:

9 $$\begin{aligned} Error^i = \left| RR_{original}^i - RR_{reconstructed}^i \right| \end{aligned}$$

Where:

- $RR_{original}^i$ is the average R–R distances for the *i*th minute in the original signal,
- $RR_{reconstructed}^i$ is the average R-R distances for the *i*th minute in the reconstructed signal,
- $Error^i$ is the absolute error for the *i*th minute.

The final heart-rate variability measurement *HV* is calculated as the mean of the errors over all the minutes.

10 $$\begin{aligned} HV = \frac{1}{N} \sum _{i=1}^{N} Error^i \end{aligned}$$

Where:

- *N* is the total number of minutes in the signal.

## Results and discussion

In this section, we present the results of our research, supported by a detailed discussion.

### Reconstruction quality evaluation

**Figure 4:**
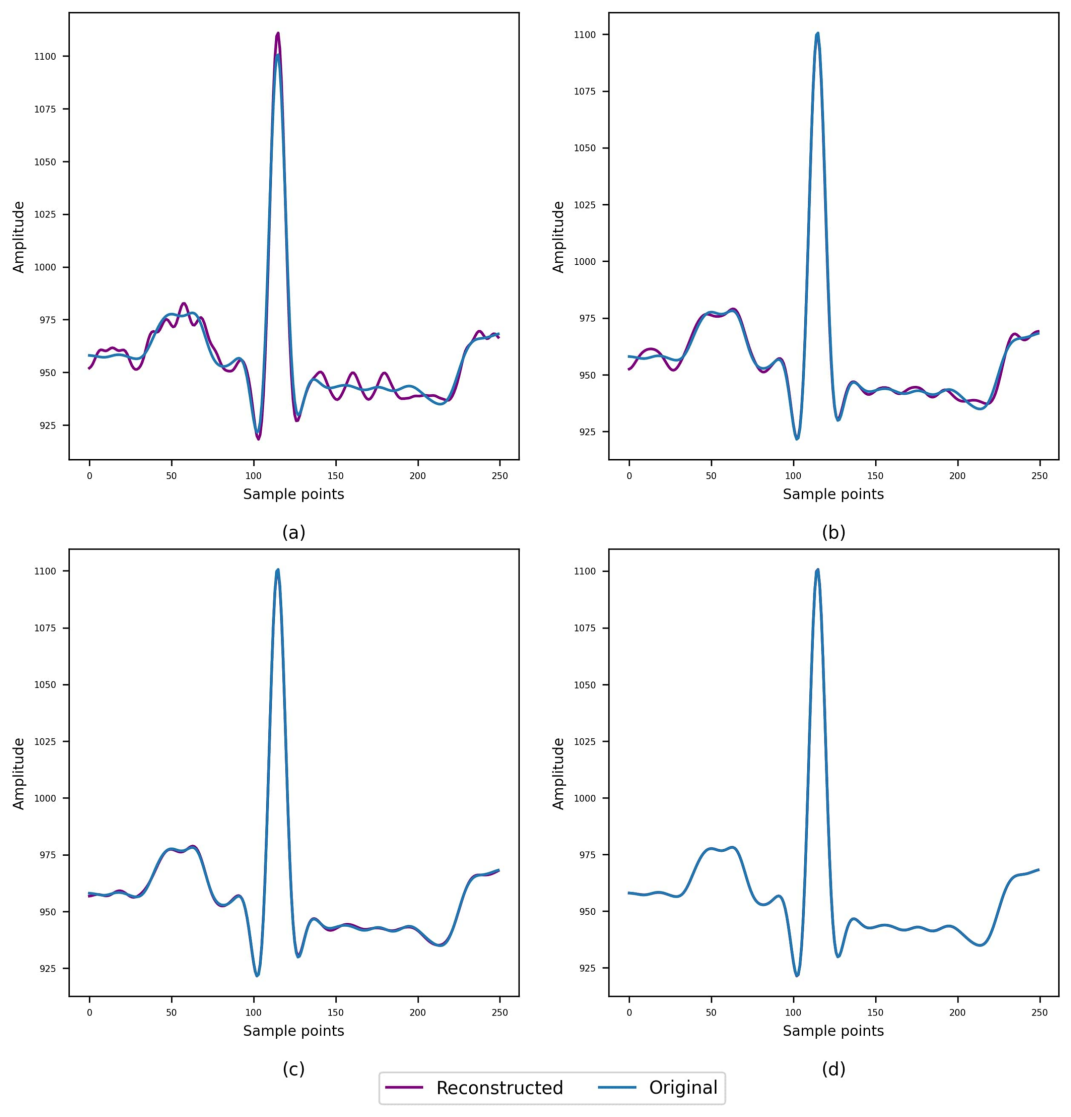
The difference between the original and signal reconstructed from the (**a**) 2-bit (**b**) 4-bit (**c**) 6-bit (**d**) 10-bit compressed signals.

Figure 4 shows the visual difference between the original and the signal reconstructed from the 2, 4, 6, and 10-bit compressed signals.

**Figure 5:**
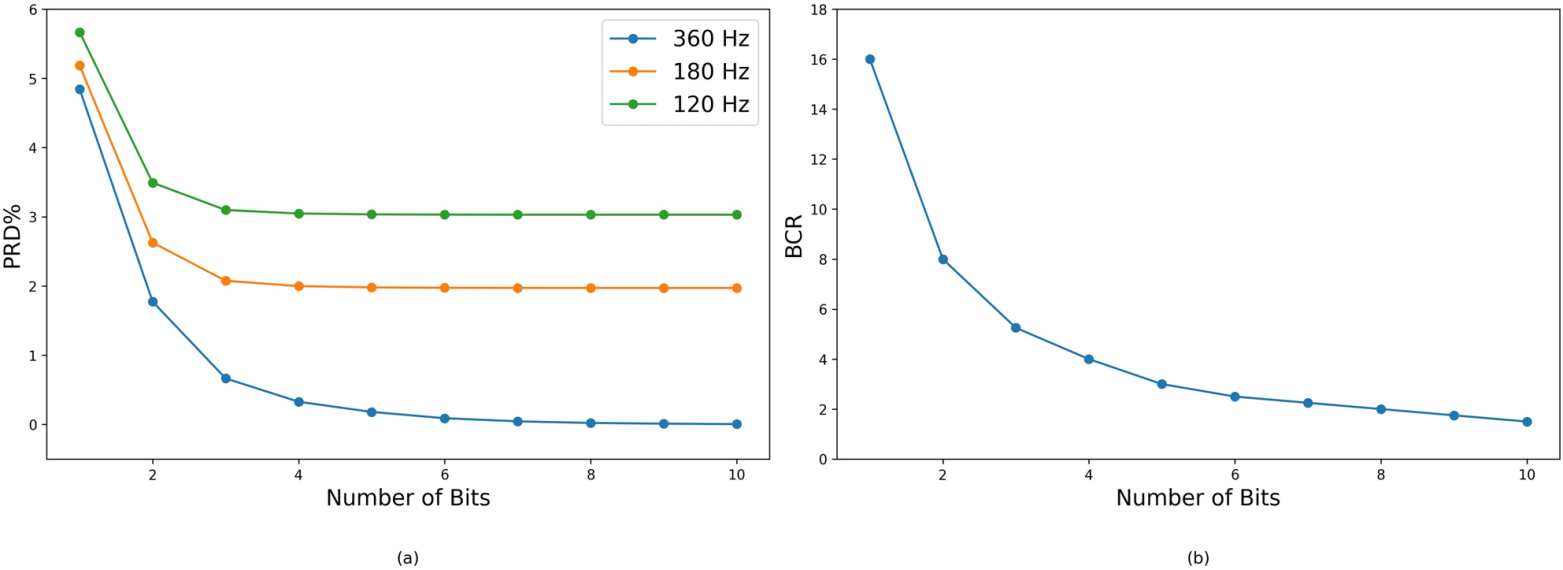
(**a**) Total error of compression using Percentage Root Means Square Error (PRD) (**b**) Bit compression ratio (BCR) variation with respect to the number of bits.

The evaluation of reconstructed signal quality relies on PRD and BCR metrics. Figure 5a shows the PRD variation with respect to the number of bits and sampling rate. For all three sampling rates, PRD remains below 5%. Notably, at a sampling rate of 360 Hz, PRD drops below 0.5% for resolutions of 4 bits and higher, achieving a nearly perfect reconstruction and preserving the original signal quality.

Figure 5b illustrates BCR variation relative to the number of bits. The combined analysis of Fig. 5a and b shows that higher BCR correlates with increased PRD, indicating a compromise in reconstructed signal quality.

Therefore, compressing original signals from an 11-bit resolution to a 4-bit resolution emerges as an optimal compromise, achieving a substantial space saving of 90.4%, a BCR of 4, and a PRD of 0.33%, giving a near-perfect reconstruction with a QS of 12. Furthermore, storing and retrieving data with a 4-bit resolution is more straightforward in a byte-aligned system compared to dealing with 11-bit data, which is an added advantage.

Table 2 shows the BCR and PRD comparison between the proposed algorithm and existing methods discussed in the related work.

### Impact on storage

The literature in this domain has primarily evaluated algorithms based on quantitative parameters like the BCR, PRD, and QS. However, they have not explicitly reported the impact on storage requirements. We believe that reporting the storage requirements for different compression levels will greatly assist users in choosing the appropriate compression level for their needs.

Table 3 shows the effectiveness of the proposed compression algorithm in terms of data reduction and space-saving. The space-saving is derived against the 155 MB size of the folder containing the original 48 records obtained from WFDB^33^.

**Table 3:** Space saving from the proposed algorithm and combined ZIP compression for 48 records compared to the original size.

Size of uncompressed 48 records of original data from WFDB^33^				155 MB
Bits	Proposed compression on 48 records (MB)	Proposed + ZIP compression on 48 records (MB)	Proposed compression space saving (%)	Proposed + ZIP compression space saving (%)
10	40.0	23.8	74.5	84.7
9	34.0	18.4	78.1	88.1
8	30.0	10.8	80.9	93.1
7	26.0	10.3	83.0	93.4
6	24.0	7.6	84.7	95.1
5	20.0	5.9	87.2	96.2
4	15.0	3.6	90.4	97.7
3	11.0	3.4	92.7	97.8
2	7.0	2.1	95.2	98.7
1	4.0	1.0	97.6	99.3

Folder sizes are reported as a collection of 48 records where each recording undergoes compression using the proposed algorithm, and multiple compressed samples are saved as 64-bit words.

Subsequently, the folder containing 48 such compressed records is stored in the ZIP file format, a widely used archive format for lossless compression. This ZIP compression, executed at a normal compression level (level 5) using the deflate method and a word size of 64, aims to highlight the cumulative impact of both the proposed algorithm and ZIP compression in terms of reducing data transmission and optimizing storage space.

It can be seen that 90.4% and higher space saving can be achieved from the proposed algorithm when compressed to 4-bit and lower resolutions. It’s noteworthy that, across all resolutions, the maximum additional space saving achieved by the more complex ZIP compression technique is approximately 15% for 8-bit resolution compared to the space-saving achieved by the proposed algorithm alone. For 4 bits and lower resolutions, this additional space saving is less than 8%. This observation demonstrates that our proposed, simpler technique effectively compresses data while utilizing minimal resources compared to the advanced ZIP compression technique.

### Computational complexity evaluation

This section studies and compares the computational cost of the proposed compression algorithm considering the number of operations, time complexity, power, and memory consumption.

Most transform-based compression techniques, such as those utilizing the Discrete Fourier Transform (DFT) or Fast Fourier Transform (FFT), exhibit higher computational complexity, typically in $O(N \log _2 N)$ or $O(N^2)$^14^. Even though low complexity algorithms with $O(N)$ complexity have been introduced specifically for wearable devices^16,25–27^, some methods still involve numerous calculations per sample^16^. While our algorithm has $O(N)$ complexity, at a more granular level, the number of operations per sample in our algorithm is detailed in Table 4.

**Table 4:** Number of integer operations per sample point.

Arithmetic operation	Number of operations
Scaling Arithmetic Operations ($\div$,$\times$)	3
Basic Arithmetic Operations (–, +, <)	6

**Table 5:** Variation of time to compress a single sample with CPU clock frequency.

Freq (MHz)	240	160	80	40	20	10
Time per sample (${\mu }$s)	0.046	0.070	0.140	0.288	0.603	1.337

Given that divisions and multiplications are more complex compared to additions, subtractions, and comparisons, we’ve segmented the operations into two groups: basic arithmetic operations (addition, subtraction, and comparisons) and scaling arithmetic operations (multiplication and division) per sample point of the proposed methodology.

The computational complexity exhibits a linear relationship with the number of samples processed. In Table 5, we present the time required for processing a single sample and the corresponding power consumption. This analysis is conducted while compressing incoming samples at a 360 Hz sampling rate across various CPU clock frequencies on the test platform. Notably, processing time demonstrates an inverse proportionality, while power consumption exhibits direct proportionality to the CPU clock frequency.

For all CPU clock frequencies, the additional power consumed by the compression algorithm fell below 0.01 mW, which is the lowest precision achievable by the multimeter. The highest processing time per sample of 1.337 microseconds was recorded at 10 MHz of CPU clock frequency. This processing time is notably small compared to the sampling rate of ECG acquisition systems.

The C program containing the compression algorithm utilizes 232 bytes of Flash memory and 4 bytes of RAM on the test platform. An added advantage is the absence of additional parameters that need tuning for optimal results from the algorithm. Furthermore, the algorithm does not contain any floating point operations. These characteristics contribute to a reduced complexity compared to existing low-complexity algorithms^16,17,25^.

### QRS complex detection performance evaluation

**Figure 6:**
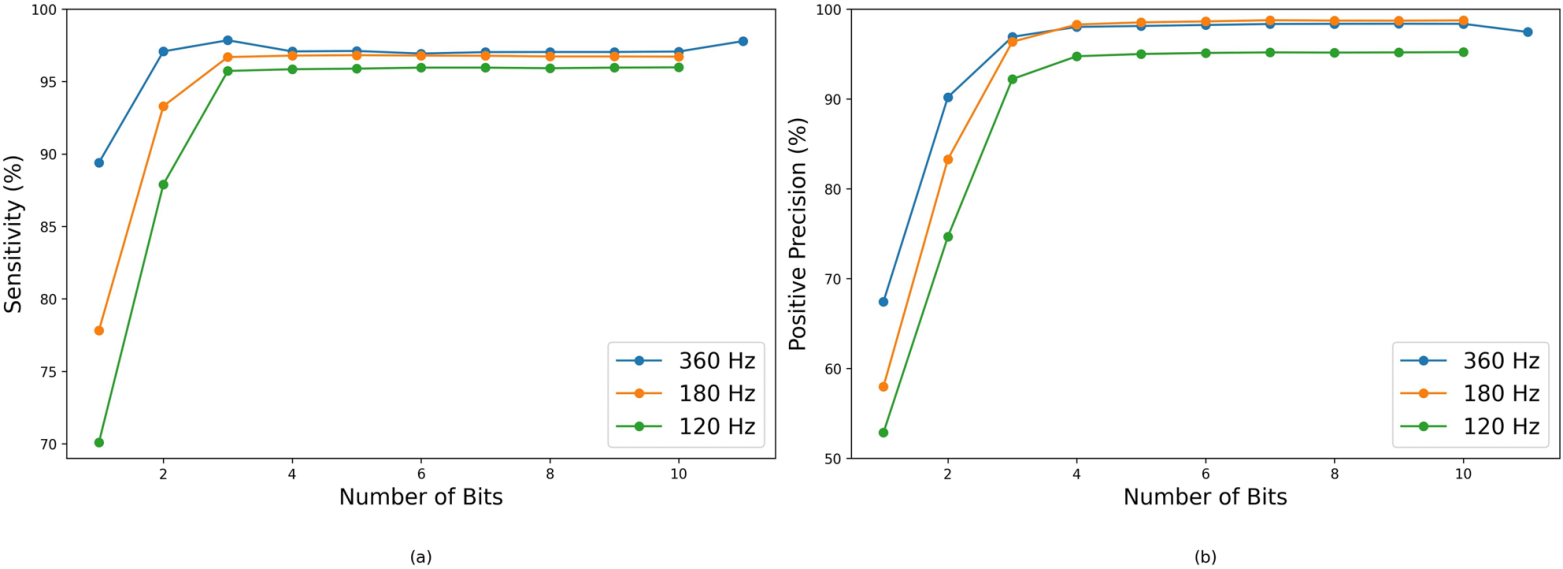
QRS peak detection (**a**) sensitivity and (**b**) positive precision performance for Pan-Tompkins Algorithm for different step sizes.

Although PRD serves as a metric for comparing how closely the reconstructed signal data points align with the original signal, it alone is inadequate for determining the suitability of the reconstructed signal in a clinical setting. To assess the practical utility of the reconstructed signal in a clinical context, the performance of QRS complex detection in terms of sensitivity and precision is evaluated at different resolutions and sampling rates, as depicted in Fig. 6a and b, respectively.

The performance of the Pan-Tompkins QRS complex detection algorithm is evaluated at different resolutions and sampling rates, as depicted in Fig. 6. The performance remains consistent for compression at 4 bits and higher resolution across the three sampling rates. The performance of the original signal at 360 Hz is indicated from the 11th bit.

Additionally, although the down-sampled compressed signal at 180 Hz shows higher error rates based on the PRD metric, it performs comparably well with signals compressed at the original sample rate of 360 Hz in terms of QRS complex detection. This implies that while the 180 Hz compressed signals may not result in perfect reconstructions, they still retain the essential features required for clinical diagnosis at 4-bit and higher resolutions.

Table 6 outlines the signal performance after reconstruction from the 4-bit resolution compressed signal using four distinct QRS complex detection algorithms. Notably, the error in the reconstructed signal’s positive precision for QRS peak detection is negative across all algorithms except for the Hamilton algorithm. This observation demonstrates that the compression not only retains crucial signal information but also enhances signal quality by eliminating unnecessary noise, leading to improved QRS peak detection performance, particularly evident in the XQRDB algorithm, which stands out as the most effective among the four algorithms.

**Table 6:** Performance of ECG signal reconstruction in detecting R peaks (Negative error: increased performance, positive error: decreased performance).

Algorithm	SE (%)			P (%)		
	Original	Rebuild	Error	Original	Rebuild	Error
Pan and Tompkins^36^	**97.79**	97.03	0.78	97.45	**98.34**	**-0.91**
Elgendi^36^	97.52	**97.60**	**-0.08**	95.91	**95.97**	**-0.06**
Hamilton^36^	**95.17**	94.98	0.20	**95.34**	95.15	0.20
XQRS^33^	99.15	**99.19**	**-0.04**	99.18	**99.23**	**-0.05**

Significant values are in bold.

Furthermore, results show that the performance reduction (if any) due to compression is negligible, emphasizing the proposed compression’s effectiveness in preserving essential features.

### Heart-rate variability performance evaluation

Heart rate variability is measured for 44 records out of the 48 MIT-BIH arrhythmia ECG records, excluding records 102, 104, 107, and 217, due to their poor performance in R-peak detection resulting from the presence of pacemakers. Table 7 displays the heartbeat variability for each record.

**Table 7:** Error of the heat-rate variability between the 11-bit original signal and reconstructed 4-bit compressed signal in a number of samples.

Record	HV	Record	HV	Record	HV	Record	HV
100	0	115	0	202	0.14	219	0.68
101	0	116	0	203	0.47	220	0
103	0.08	117	0	205	0	221	1.7
105	0.04	118	0	207	4.92	222	0.15
106	0.21	119	0.03	208	6.47	223	0.05
108	3.61	121	0	209	0	228	0.11
109	0	122	0	210	0.67	230	0
111	0	123	0.11	212	0	231	0
112	0	124	4.51	213	0.02	232	0.45
113	0	200	0.1	214	0	233	0
114	3.45	201	0.1	215	0	234	0

Heart-rate variability measurement quantifies how much the average R–R distance changes in samples within a 1-min window. Across the majority of records, this variation is nearly zero, indicating that there is no change in R–R distance measurements. The maximum change observed is 6.47 samples, which is a 0.03% change compared to the 21,600 samples in 1-min. This implies that the compression algorithm doesn’t significantly affect the overall morphology of the signal.

### Performance validation on the INCART database

As discussed earlier, the optimal number of bits for compression has been identified as 4 bits. The results presented in Table 8 were obtained by compressing a single channel of all 75 recordings in the INCART database, originally at 16-bit resolution, down to 4 bits. The data shows that the algorithm achieves a PRD of less than 1% while maintaining a BCR of 4, even when compressing higher-resolution signals to 4 bits. This demonstrates that the reconstructed signal is nearly perfect with minimal distortion, achieving a 91.5% space saving compared to the original 16-bit recordings.

These results highlight the robustness of the algorithm, as it maintains its performance regardless of artefacts present in different ECG-acquiring systems.

**Table 8:** Performance of the proposed algorithm on 75 records of the 16 bit resolution INCART database compressed to 4 bits.

Size of uncompressed 75 records of original data from WFDB^33^					195 MB
BCR	PRD (%)	Proposed compression on 75 records (MB)	Proposed + ZIP compression on 75 records (MB)	Proposed compression space saving (%)	Proposed + ZIP compression space saving (%)
4	0.98	16.50	2.6	91.5	98.7

### Application and impact

The novel ECG compression algorithm discussed in this paper presents a significant advancement in cardiac monitoring for healthcare. Its simplicity and low computational demands make it suitable for real-time monitoring through wearable devices, enabling continuous, long-term tracking of cardiac health. This advancement aids in the early detection and management of heart conditions by allowing quick and accurate analysis of ECG data, preserving crucial diagnostic information for enhanced arrhythmia detection and heart rate variability analysis.

Financially, this innovation reduces ECG data management costs by minimizing storage and transmission requirements, thus making healthcare solutions more affordable, particularly in remote monitoring scenarios. Moreover, its integration into wearable devices without the need for specialized hardware further enhances its economic viability and accessibility, benefiting resource-constrained healthcare settings.

The algorithm’s application extends beyond individual care to research and population health management. The algorithm facilitates the efficient processing and analysis of large volumes of ECG data from clinical trials, serving as a plug-and-play solution with readily measured power and time measurements. This accelerates research in cardiology and supports advancements in treatment protocols. Healthcare organizations can harness the compressed ECG data for population health management and predictive analytics, enhancing both individual patient care and overall health outcomes at the population level.

Improved cardiac care and diagnosis can lead to better patient outcomes, reduced hospital readmissions, and an overall enhancement in quality of life. Additionally, the algorithm’s reduced data storage and transmission needs contribute to lower energy consumption in healthcare Information Technology (IT) infrastructure, aligning with the need for environmentally sustainable medical technologies. Thus, the algorithm offers benefits across various domains, from patient care to environmental sustainability, while also being economically advantageous for healthcare systems.

Although this algorithm efficiently compresses ECG signals, it doesn’t rely on specific characteristics of the ECG signal for compression. Even if initially designed for ECG classification, it holds promise for extending its utility to compressing signals beyond ECG.

## Conclusion

The proposed hybrid PWM-quantization compression algorithm requiring nine fixed-point arithmetic operations, containing only three divisions and multiplications per sample point, strikes a balance between simplicity, compression ratio, and signal fidelity. Achieving a bit compression ratio of 4 with a 90.4% space saving when multiple samples are saved as 64-bit words, the proposed algorithm demonstrates nearly perfect reconstruction on the MIT-BIH ECG database for resolution as low as 4 bits per sample, preserving critical signal characteristics for accurate arrhythmia detection, heart-rate variability calculations, and ECG waveform diagnosis algorithms. The algorithm’s simplicity is further emphasized by its lack of parameter tuning and avoidance of floating-point calculations. Evaluation of signal quality using PRD, BCR, QS, and QRS complex detection ability from existing detection algorithms confirms its effectiveness in real-world situations. As a result of the low resource consumption and simplicity, this algorithm can be implemented on wearable devices to store and transmit data efficiently for long-term monitoring applications such as arrhythmia classification.

The proposed compression algorithm offers a plug-and-play solution characterized by low complexity, minimal power consumption, and minimum processing delays. Thorough testing in cardiac assessment applications validates its effectiveness, particularly in achieving noteworthy results for multiple QRS detection algorithms and heart-rate variability measurement, making it accessible and accurate for widespread use.

## Data Availability

The datasets analyzed during the current study are available in the PhysioBank repository: $\bullet$ MIT-BIH arrhythmia dataset: https://www.physionet.org/content/mitdb/1.0.0/. $\bullet$ INCART dataset: https://www.physionet.org/content/incartdb/1.0.0/.
